# LFP polarity changes across cortical and eccentricity in primary visual cortex

**DOI:** 10.3389/fnins.2023.1138602

**Published:** 2023-02-27

**Authors:** Fereshteh Khodaei, S. H. Sadati, Mahyar Doost, Reza Lashgari

**Affiliations:** ^1^Department of Mechanical Engineering, K. N. Toosi University of Technology, Tehran, Iran; ^2^Institute of Medical Science and Technology, Shahid Beheshti University, Tehran, Iran

**Keywords:** receptive field, V1 neurons, LFP mapping, current source density, sink-source, laminar organization, eccentricity, LFP polarity

## Abstract

Local field potentials (LFPs) can evaluate neural population activity in the cortex and their interaction with other cortical areas. Analyzing current source density (CSD) rather than LFPs is very significant due to the reduction of volume conduction effects. Current sinks are construed as net inward transmembrane currents, while current sources are net outward ones. Despite extensive studies of LFPs and CSDs, their morphology in different cortical layers and eccentricities are still largely unknown. Because LFP polarity changes provide a measure of neural activity, they can be useful in implanting brain-computer interface (BCI) chips and effectively communicating the BCI devices to the brain. We hypothesize that sinks and sources analyses could be a way to quantitatively achieve their characteristics in response to changes in stimulus size and layer-dependent differences with increasing eccentricities. In this study, we show that stimulus properties play a crucial role in determining the flow. The present work focusses on the primary visual cortex (V1). In this study, we investigate a map of the LFP-CSD in V1 area by presenting different stimulus properties (e.g., size and type) in the visual field area of Macaque monkeys. Our aim is to use the morphology of sinks and sources to measure the input and output information in different layers as well as different eccentricities. According to the value of CSDs, the results show that the stimuli smaller than RF’s size had lower strength than the others and the larger RF’s stimulus size showed smaller strength than the optimized stimulus size, which indicated the suppression phenomenon. Additionally, with the increased eccentricity, CSD’s strengths were increased across cortical layers.

## 1. Introduction

One of the critical elements of central visual processing is the integration of local signals that are part of visual events broken by retinal cells (Kim et al., 2015). Local field potentials have been reported in the neocortex, subcortical structures such as the striatum, thalamus, and other regions including the basal ganglia (Tanaka and Nakamura, 2019). V1 is assumed to be similar to laminar cortical circuitry in other cortical regions (Schroeder et al., 1998). LFP compared with spiking activity is easy to record and it could be used in neural prostheses (Andersen et al., 2004; Pesaran et al., 2006; Berens et al., 2008). A number of researchers worked on the horizontal and vertical spread of LFP in the cortex, trying to detect how many neurons LFPs originate from Schroeder et al. (1992), Wang et al. (2005), Kreiman et al. (2006), Katzner et al. (2009), and Xing et al. (2009).

Cell types and projection patterns fundamentally differ in a cortical laminar organization (Maier et al., 2010). Different cortical layers are activated as a result of integration of information and dissociation at particular times (Mahmud et al., 2017). Kim et al. (2015) examined the subthreshold local field potential in surrounding interactions in V1 of the awake monkey. The cortex has distinct intralaminar and interlaminar connectivity patterns. The laminar structure of the cerebral cortex is a usual anatomic shape in the brain and cortical layers have different functional properties (Leventhal et al., 1995; Martinez et al., 2002; Buffalo et al., 2011; Goense et al., 2012; Bijanzadeh et al., 2018). V1 is known to have six layers with different response characteristics and connection structures. V1 output layers have strong horizontal and feedback connections (Wang et al., 2020). The major sources of LFP activity are broadly synaptic inputs to local cortical regions (Martín-Vázquez et al., 2018). LFPs are either under the influence of nonspecific effects like a state of excitability or continuous cortical activity, and this cortical activity could be spontaneous or evoked by a preceding stimulus (Schaefer et al., 2015). Roerig and Chen (2002), Gawne (2010) reported LFP’s positive polarity referred to inhibitory information which is the result of presenting a stimulus to the receptive field (RF) surrounding. Lashgari et al. (2012) analyzed LFP and single-unit activity for different frequencies in response to different stimulus features like orientation, contrast, size, temporal frequency, and even spatial phase.

Action potentials, excitatory currents, and inhibitory currents are sources from which LFPs originate from. Each of these sources may contribute to the induction of LFPs differently. For example, one of the sources that contribute little to signal generation is GABAA (Kim et al., 2015). Recent advances have made it possible to simultaneously record very large populations of neurons to decode underlying activity. The result of studies on the laminar circuitry of the primary visual cortex shows a complex map of anatomical connectivity between the V1 layers (Dougherty et al., 2017). The shape of individual LFPs carries helpful information about the underlying neuronal network (Mahmud et al., 2017).

It is well known that the CSD shows the location of sinks and sources. Buzsáki et al. (2012) state that the contractual definition of sinks and sources was as follows: sinks are sites on the neuronal membrane where positive charges enter the neuron by convention, and sources are the locations along the neuronal membrane where positive charge flows out of the neuron. Sotero et al. (2015) recorded LFP using laminar probes of area S1 of the rat brain and computed current source density and intra- and inter-laminar phase-amplitude coupling of CSD for all possible pairs. Martín-Vázquez et al. (2018) also performed current source density analysis. Single LFP recordings demonstrate inhibitory and excitatory synaptic activities. LFPs cannot provide exact spatial information such as the location of layers or depth. The CSD is based on the second spatial derivative of the field potentials along with radial depth. This analysis provides precise spatial and temporal information about the synaptic activity (sinks), and therefore implies the mechanism of their generation and detection of the neuronal information flow (Schaefer et al., 2015).

Dougherty et al. (2017) tested sensory stimulation dependency on laminar cortical interactions between the alpha cycle and spiking fluctuations. Different combinations of layer-specific inhibition and excitation are reflected by the functional properties of cells in different V1 layers (Bijanzadeh et al., 2018). Most studies have shown that the sink in V1 corresponds most closely to layer 4Cα (Mitzdorf and Singer, 1979; Schroeder et al., 1991), but (Maier et al., 2010) showed it is associated to the middle layers. Since infragranular layers are the primary target of cortical feedback projections, the control of these layers on neuronal excitability across the laminar cortical column is a fascinating hypothesis (Dougherty et al., 2017). Mahmud et al. (2017) overviewed some of the available neuronal probes, the neuronal signals recorded, and a few automated methods to analyze the acquired LFPs. Tanaka and Nakamura (2019) showed that focal excitatory input can generate LFPs on the order of 0.1 mV in areas without laminar structure. Gieselmann and Thiele (2022) separate laminar spectral signatures of feedforward and feedback signals communication using different stimulus sizes in the primary visual cortex of monkeys. LFPs (linear superposition of the electric potentials) are generated by separated sinks and sources. It is believed that in producing LFPs, current sinks and sources spatially separated in between the apical and basal dendrites are necessary (Tanaka and Nakamura, 2019).

Using CSD, (Yang et al., 2022) tried to figure out how the primary visual cortex would process surface luminance information across its different layers. Wang et al. (2020) presented stimulus orientations in the primary visual cortex and measured laminar-specific responses with CSD analysis. Inhibition and excitation are coordinated by mechanisms that are unclear. They generate functions in the six layers of the cortex. In CSD profiles, current sources set forth to show mostly the passive return currents. Moreover, sinks show excitatory occurrences like synaptic activations (excitatory or inhibitory) and axonal depolarizations (Schaefer et al., 2015). van Kerkoerle et al. (2014) saw a current sink in layer 4C and a current source in the deep layers at the CSD profile which coincided with the onset of the visual response in the multiunit activity (MUA). Kajikawa and Schroeder (2011) compared LFP, current source density (CSD), and MUA signals in the auditory cortex. Bijanzadeh et al. (2018) analyzed LFP using CSD. They showed that the earliest activity extracted by stimuli centered on the receptive field occurs in layer 4C.

In the studies conducted on LFP and CSD resulting from it, suppression phenomenon is not mentioned. The studies conducted on this issue have described and explained suppression using spike. Knierim and van Essen (1992) studied macaque monkey V1 response to textured patterns using suppression index. In Nothdurft et al. (2000) investigated the response profiles to texture border patterns in area V1 using firing rate. Webb et al. (2005) investigated surround suppression in striate cortex of macaque with suppression index. Petrov and McKee (2006) studied contrast sensitivity using suppression factor. Nurminen and Angelucci (2014) analyzed components of surround modulation in V1 using spike rate. Also in these studies, the effect of suppression on the layers has not been investigated and tested.

Henry et al. (2013) considered laminar differences using spike rate to characterize the extraclassical receptive field in V1. Bijanzadeh et al. (2018) studied laminar latencies in primary visual cortex of monkey. They investigated the suppress phenomenon by varying the stimulus size.

In the present study, laminar electrodes were used to record LFPs. Using CSD analysis, the changes in sinks and sources, including the strongest and first ones in terms of the amount of occurrence in layers, were examined here. One of the most important features and advantages of layered electrodes is that they can be used to compute current source densities. CSD analysis is a well-analytic technique for computed microscopic current sinks and sources in the extracellular medium area (Mitzdorf and Singer, 1979, Mitzdorf, 1985). As mentioned, in the RF studies conducted on suppression, none of them used CSD. We tried to study this phenomenon by using LFP and the resulting CSD.

## 2. Materials and methods

### 2.1. Surgical procedures

Macaque monkeys (Macaca fascicularis) were quarantined for 6 weeks after purchase and then kept in groups. In total, signals were recorded from 4 monkeys (2 male and 2 female). The data of 7 penetrations, which were completely vertical in V1, were used in this work.

All experiments were in accordance with protocols approved by the University of Utah Institutional Animal Care and Use Committee and with NIH guidelines.

Monkeys were first anesthetized with ketamine (25 mg/kg, i.m.), and then anesthesia was maintained with isoflurane (2%). Vital signs including body temperature, electrocardiogram, oxygen level and blood pressure and lung pressure were monitored during surgery.

### 2.2. Electrophysiological recordings

Data were made in parafoveal V1 (2–6 Degree eccentricities), collected with 30 kHz sampling rate and amplified using a 128 channel system (Cerebus, 16-bit A-D, Blackrock Microsystems, Salt Lake City, UT, USA). The electrode used was 24-channel linear arrays (100 mm inter-contact spacing, 20 mm contact diameter; V-Probe, Plexon, Texas). The raw voltage recordings were band-pass filtered (1–100 Hz, 2nd-order Butterworth filter) and down sampled to 2 kHz to obtain LFPs. These data have already been used in the article of Bijanzadeh et al. from the perspective of latency (Bijanzadeh et al., 2018).

### 2.3. Visual stimuli

The stimuli were generated with the below MATLAB, monitor, and ViSaGe controlled system properties:

• MATLAB (Mathworks Inc., Natick, MA, USA; RRID:SCR_001622).
• CRT monitor (Sony, GDM-C520K, 600*800 pixels, 100 Hz frame rate, mean luminance 45.7 cd/m2, at 57 cm viewing distance).
• ViSaGe system (Cambridge Research Systems, Cambridge, UK; RRID:SCR_000749).

**FIGURE 1:**
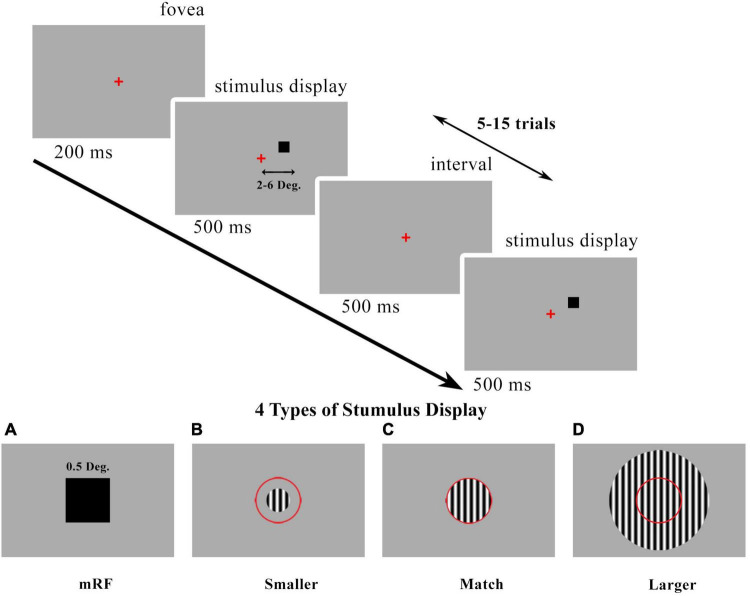
Visual presentation timeline. Local field potentials (LFPs) were recorded in V1 cortical column (2°−6° eccentricities). The desired stimulus is presented at 500 milliseconds, 5–15 trials, and interleaved with 500 milliseconds. **(A)** 0.5° black square stimulus centered on the columnar minimum receptive field (mRF). Drifting grating patch stimulus with changes in size [smaller than receptive field (RF) **(B)** match stimulus size with RF **(C)** and larger than RF **(D)**]. The red circle is a schematic view of the RF. We called these drifting grating types of stimuli as summation RF (SRF). Red present the center of fovea.

The black square stimulus and the optimized grating stimulus are two types of stimuli that were investigated in this research. The stimulus presentation timeline and stimulus types are shown in Figure 1. The first type stimulus was a 0.5 degree black square centered on a columnar RF for all eccentricities. In the second type of stimulus, which was grating, three different modes of stimulus smaller than the receptive field, stimulus with the match size with the receptive field, and stimulus with a larger size than the receptive field were investigated for all eccentricities. The size of them according to stimulus type and eccentricity is shown in Table 1.

**TABLE 1:** Size of stimuli in all eccentricities.

Ecc. (Deg.) Stimulus Diameter (Deg.)	2.5–3	3	4.1	4.1–4.3	5	5.2	5.4
mRF	0.5	0.5	0.5	0.5	0.5	0.5	0.5
Match	0.4	0.4	0.6	0.8	0.8	0.8	0.6
Larger	0.8	0.8	1.2	1.5	1.5	1.5	1.2
Smaller	0.2	0.2	0.2	0.2	0.2	0.2	0.2

The size of the stimulus according to its type and eccentricity is stated in this table. All values are in degrees.

### 2.4. Layers alignment

Current source density analysis and histological observation have been used in this study to localize LFP signals to a specific layer in V1.

### 2.5. RF mapping

**FIGURE 2:**
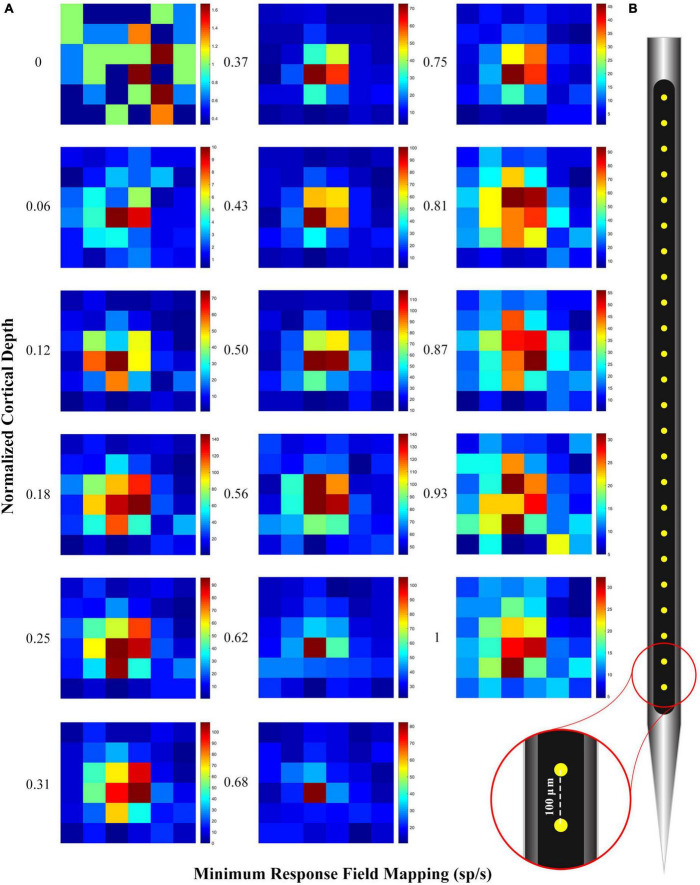
**(A)** Receptive field (RF) mapping plot with spike per second in normalized cortical depth. The heatmap was obtained from the average of multiunit activity (MUA) in response to the 0.5 degree black square stimulus that was randomly presented in all 36 squares (Each square shown in the figure is 0.5 degrees). A good alignment of the RF mapping across the contact points indicates that the electrode is perpendicular. **(B)** 24-channel linear electrode arrays (100 μm inter-contact spacing, 20 μm contact diameter; V-Probe, Plexon, Texas).

At first, the approximate RF location of neurons in each V1 cortical column has been identified manually. Then, by presenting 0.5° black squares in a 3°*3° visual field on the approximate RF location, the accurate RF location of neurons in each cortical column was identified. Figure 2 shows an example of RF mapping along the electrode. The heatmap drawn in this figure shows the perpendicularity of the electrode in the tissue. To confirm the verticality of the electrode, we plotted the spike response rate to the stimulus presentation in each of the 36 considered square grids as a heatmap. As can be seen, the location of the RF is correctly specified.

### 2.6. Current source density

A black square of 0.5° flashed over a 3°*3° visual field area. LFP recordings were made in V1 (2°−6° eccentricities; seven penetrations) using 24-channel linear electrode arrays (100 μ*m* inter-contact spacing, 20 μ*m* contact diameter; V-Probe, Plexon, Texas). The stimulus presented in the receptive field of these 36 blocks at 500 milliseconds, 5–15 trials, and interleaved with 500 milliseconds. The location of the RF was found by presenting the stimulus in 36 squares. In our analyses, we used the LFP results in response to the black square stimulus presented in the RF. The type of stimulus was then changed and the investigations were repeated. Drifting grating patch of increasing size centered over the aggregate mRF of the column using 100% contrast presented at the optimal parameters for neurons. Three types of stimuli were analyzed based on their sizes, including smaller than the receptive field, match stimulus size with RF, and larger than that.

Current source density was applied to the response of LFPs resulting from the presentation of stimuli. Current source density (CSD) was applied to the LFP in this study, and in particular, the kernel CSD was used. It is known that CSD is the second spatial derivative of the LFP signal, as shown in Equation 1. The CSD was baseline corrected (Z-Scored).


(1) $$C\,S\,D\,\left(x\right)=-\sigma \,\frac{L\,F\,P\,\left(x-h\right)-2\,L\,F\,P\,\left(x\right)+L\,F\,P\,\left(x+h\right)}{h^{2}}$$

Where *LFP* is the Voltage (μ*V*), *x* is the point at which CSD is calculated, *h* is the recording contacts spacing (here 100 μm), and σ is the cortical tissue conductivity [0.4 Siemens per meter (S/m)]. Siemens per Meter (S/m) is a unit in the category of Electric conductivity. This unit is commonly used in the SI unit system. (S/m) has a dimension of M^–1^L^–3^T^3^I^2^, where M is the mass, L is the length, T is the time, and I is the electric current.

As many as seven penetrations were examined here, ranging from 2.5 to 5.4 degrees of eccentricity. After extracting the CSDs for these penetration points, the peaks and troughs of the CSDs were then determined by using the MATLAB software and writing the appropriate MATLAB script. These points represent sources and sinks, respectively. CSDs are second spatial derivatives of LFPs. CSD were interpolated every 10 mm to estimate CSD across layers. The signs of the resulting CSDs show the direction of the concavity of the signals. By extracting local maxima and minima (value and time of occurrence) and filtering out small values, CSD peaks and troughs are obtained.

## 3. Results

We used CSD analysis to investigate the input and output of information according to the size of the stimulus relative to the receptive field with increasing eccentricity. It is noted that the strongest sinks and sources were selected for analysis, and the rest of the points obtained were removed.

**FIGURE 3:**
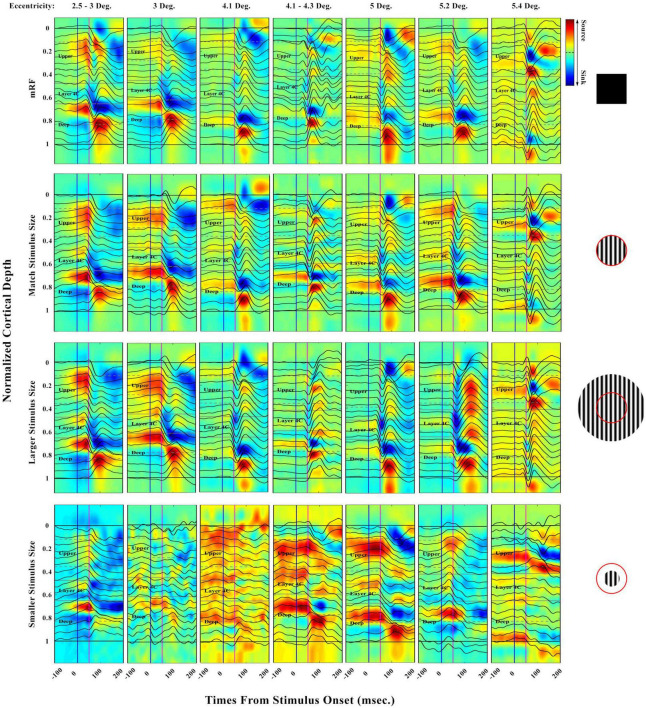
Laminar evoked local field potential (LFP) profile and distribution of extracellular current sinks and sources (CSD) obtained in response to four types of stimuli: [0.5 Deg. black square, match, larger, and smaller stimulus size with respect to receptive field (RF)]. The **(first row)** shows the response of the LFPs and CSDs to the black square stimulus of 0.5 degrees in different eccentricities. The **(second row)** shows the response to the stimulus with an equal size to RF. In the **(third row)**, the response to the stimuli with a larger size, and in the last row, the response to the stimuli with a smaller size than the RF are presented. The vertical blue line refers to the onset and the pink one to 50 msec. Horizontal solid black lines indicate the boundary of the beginning and the end of the layers. The horizontal gray lines refer to the boundary of the layers. The vertical axis shows the normalized cortical depth, and the horizontal axis represents time. The time axis indicates a range of –100 to 200 milliseconds. LFP recordings were made in V1 at 2°–6° eccentricities and seven penetrations. The exact value of the eccentricity of each penetration is indicated at the top of the figure. The red circle is a schematic view of the RF. Sinks can be distinguished by blue color and sources by red. The shape color guide is placed on the left side and above the figure.

The strength and time parameters of the sinks and sources obtained as well as the layers were investigated. The effect of an increase in eccentricity was also studied. It is clear that in order to investigate the result of an increase of eccentricity on the strength and time of sinks and sources, each layer during the increase of eccentricity should be examined separately due to the difference in the physiological nature of the layers (input and output). For future studies, it is recommended that this investigation be experimented and analyzed with eccentricity increases for each layer separately. Evoked LFP profile and their CSD analysis can be seen in Figure 3. The laminar distribution of LFPs and corresponding CSDs are shown with increasing eccentricities. Different cases for a range of 2°–6 eccentricities in the primary visual cortex were analyzed, with the ensuing results presented in appropriate diagrams. In these diagrams, the boundaries of the layers are defined so that the upper, middle, and deep layers can be clearly distinguished.

Except for the response to the stimulus smaller than RF, which does not have a specific information, in the other three types of stimuli in all eccentricities, the primary sink appears in layer 4C, which of course has different strengths. The primary sink is weaker in the deep layers than in the middle layers. Primary sources can be seen in layers 2/3, and 5. The strengths of these sources are greater in the small eccentricities than in the large ones. Secondary sources are seen in the middle and deep layers. It should be noted that the secondary source is visible and strong in deep layers. Due to the LFP responses in each specific eccentricity, we can see that the time interval between the sink and the sources (compression of curve fluctuations) is decreasing in the stimulus mode equal to the RF, black square, and larger than RF, respectively.

In the CSD diagram, the blue color represents the sinks and the red color represents the sources. In response to the black square stimulus, we can see the more yellow CSD with increasing eccentricity in total sampling time. Of course, it should be noted that with increasing eccentricity, the receptive field becomes larger and the size of our stimulus is still a half-degree black square. Perhaps the reason for the decrease in information input is the small size of the stimulus compared to the size of the receptive field of that area. But by observing the CSD in the two stimulus modes equal to the RF and larger than RF, we see that the total amount of sinks or information input decreases and the CSD graph turns yellow. The response to the stimulus smaller than RF is very different from that related to the other categories in terms of appearance. The LFP diagram shows that the concavity is not visible. In other words, the input of information in the circular stimulus smaller than the RF is not significant. The CSD diagram is also drawn for all stimuli. As can be seen, in stimuli smaller than RF, the input and output of the information content are rather weak.

**FIGURE 4:**
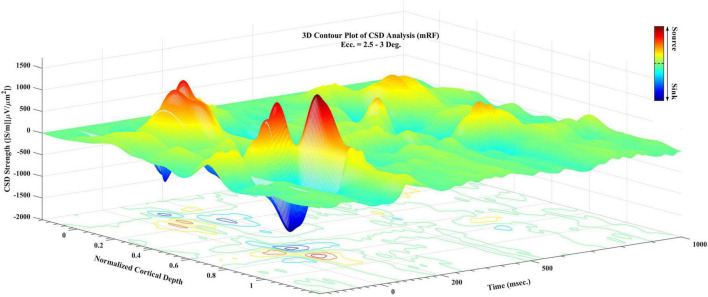
A 3D contour plot of current sinks and sources (CSD) during visual stimulation. Cortical depth is normalized for better understanding. Sampling time in all recording times is presented. The third axis shows the CSD value, with the positive values referring to sources and negative values to sinks. The guide bar explains the colors of the chart.

In Figure 4, a 3D contour plot of CSD is shown. This 3D diagram is presented for a better understanding of how to extract sinks and sources. As demonstrated in this diagram, there are peaks and troughs in the graph. The red peaks represent the sources and the blue troughs represent the sinks. The characteristics of the largest sinks and sources were extracted, and the time related to the peak of each of these points as well as their magnitudes and which layer they are located in have been listed for analysis.

**FIGURE 5:**
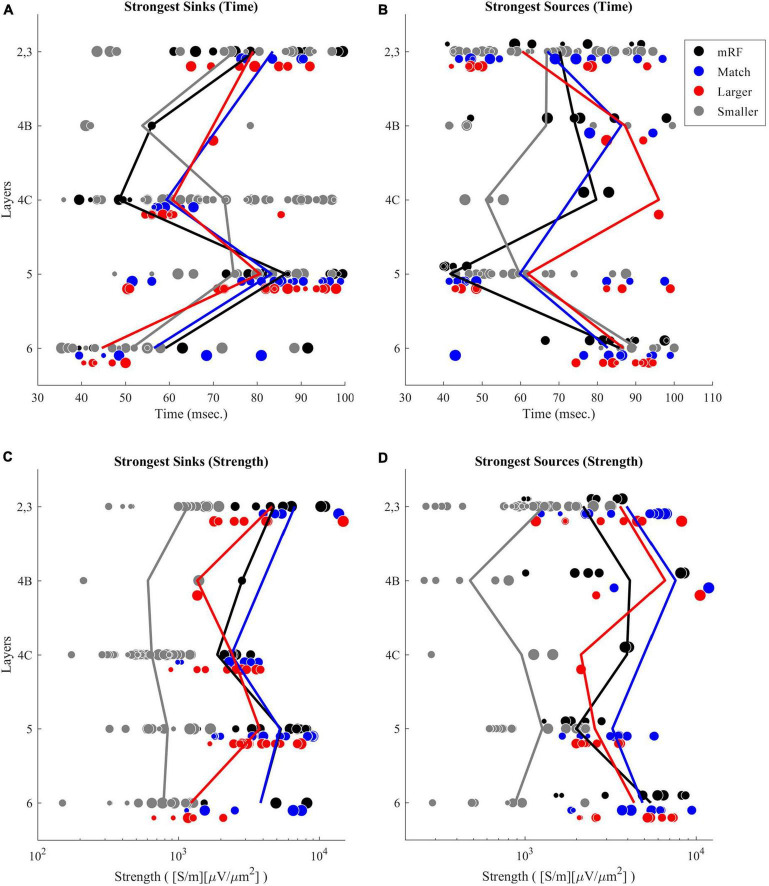
Time of strongest sinks **(A)** and sources **(B)** by differentiating the layers. Black dots refer to 0.5° black square stimulus. Blue, red, and gray ones refer to stimuli of equal size with receptive field (RF), larger, and smaller than RF, respectively. The solid lines also show the mean value of times for each stimulus. The strength of the strongest sinks **(C)** and sources **(D)** by differentiating the layers are also presented. The solid lines also show the mean value of strength for each stimulus. In the panels **(A,B)**, the horizontal axis shows the time. In the panels **(C,D)**, the horizontal axis shows the strength of current sinks and sources (CSD). The vertical axis in all figures defines the layers. The chart guide differentiates each stimulus based on the specified color. The size of the marked points on the graph increases with increasing eccentricity.

You can see the time of the most powerful selected sinks and sources in Figures 5A, B and the strengths of these sinks and sources by layers in Figures 5C, D. In Figures 5A, B, the dots indicate the time for the sink and source in each layer for all penetrations recorded. The size of the points increases with increasing eccentricity. As can be seen, the first sinks occur in layers 4C and 6, which are the input layers. This means that the beginning of the incoming flow (sink) occurs earlier in the two layers 4C and 6. Likewise, the fastest sources also occur in layers 2/3, and 5, indicating the outcoming flow of information. In fact, we can see a projection of information entering layers 4C and 6, and exiting from layers 2/3, and 5. The projection of information input from the rest of the layers can also be seen at later times. The output of information can be seen in all layers. These results also show the strengths of these sinks and sources in Figures 5C, D. Analyses related to extracting the features of the largest sinks and sources, including their occurrence times and values were performed. In addition, the values of the peaks and troughs corresponding to sources and sinks up to 60% of the largest ones were also extracted.

In Figures 5C, D, the weakest sinks and sources are seen in the stimulus type smaller than the receptive field. The sinks and sources corresponding to the black square stimulus are weaker than that related to the stimulus equal to the receptive field size. A notable interesting point that can be seen is that the sinks and sources related to the stimulus larger than the receptive field are weaker than those from both the equal stimuli and the black square stimuli. In fact, the phenomenon of suppression is clearly seen.

Comparing the strength of the sinks in the layers reveals that the sinks of layers 2/3, and 5 are stronger than those of layers 4C and 6. Since the sinks of layers 2/3, and 5 occur later than those of layers 4C and 6, this result indicates that we have stronger sinks over time. Regarding the strength of the sources, a result with similar pattern can be observed in Figure 5D. The sources of layers 5, 2, and 3 are weaker than the sources of layers 4B and 6 because they occur earlier.

Since the distribution of the collected data was not large in terms of number and did not have a normal distribution, based on this, to compare the time and strength of occurrence of the strongest sinks and sources among different layers of the cortex, the Kruskal–Wallis non-parametric test was used. The test was equivalent with a one-way ANOVA test.

**FIGURE 6:**
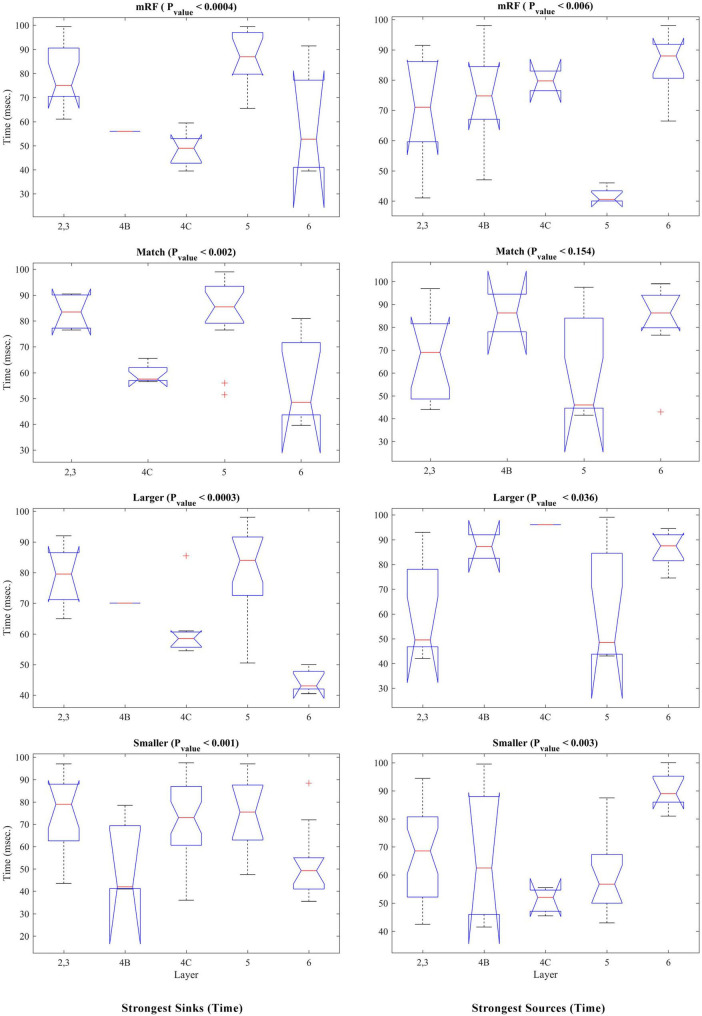
Kruskal–Wallis test of strongest sinks and sources from the perspective of times of occurrence. The **(left, right)** columns refer to the sink and source time tests, respectively. Each row shows a type of stimulus indicated above it. The number of layers is specified on the X-axis and the Y-axis shows the time in milliseconds. At the top of each figure, the type of stimulus and the *P*_value_ of the statistical test are written.

Kruskal–Wallis statistical test was performed to check the occurrence time of sinks and sources based on the type of stimulus. Figure 6 shows the results of this investigation. The left column corresponds to the time of the sinks and the right column corresponds to the time of the sources. Each row represents a stimulus type. The *P*_value_ obtained show that in all types of stimulus, there is a significant difference between the layers in terms of the time of occurrence of sink and source (except in match stimulus size with RF for source time).

**FIGURE 7:**
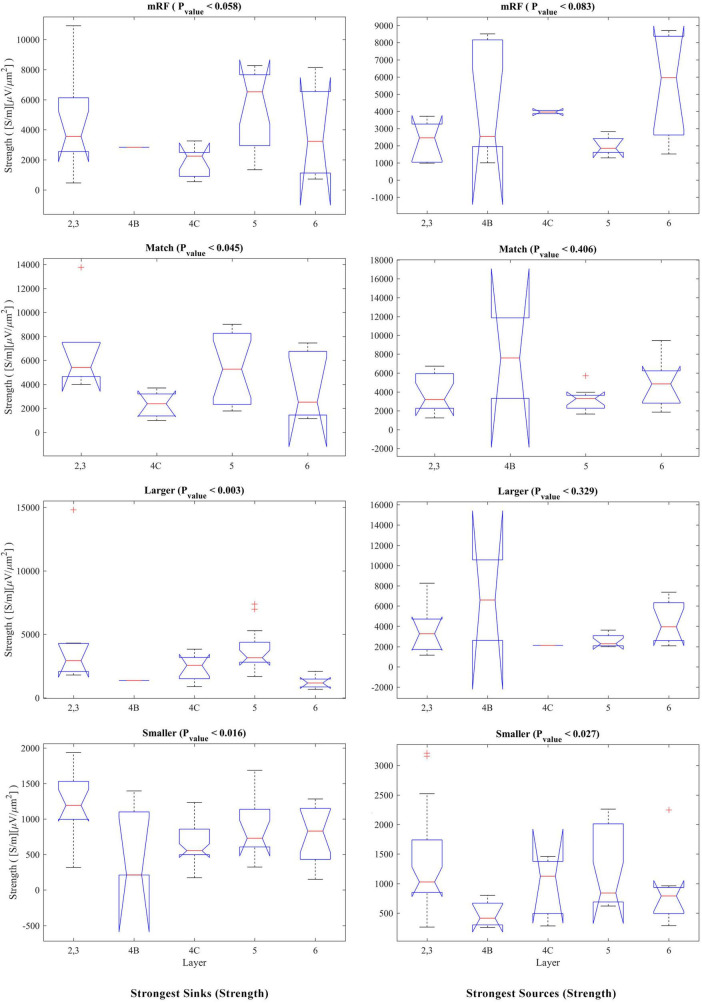
Kruskal–Wallis test for the strongest sinks **(left column)** and sources **(right column)** strengths by differentiating the layers. In the upper part of each diagram, the type of stimulus and the *P*_value_ related to the Kruskal–Wallis test are indicated.

The statistical analysis of the strength of sinks and sources separately for each stimulus and on the layers is presented in Figure 7. The left column shows the sinks. In the right column, the result of checking the sources is shown. In the match stimulus size with RF for the sink, in the smaller than RF for the sink and source, and in the larger than RF for the sink, the *P*_value_ is significant.

**FIGURE 8:**
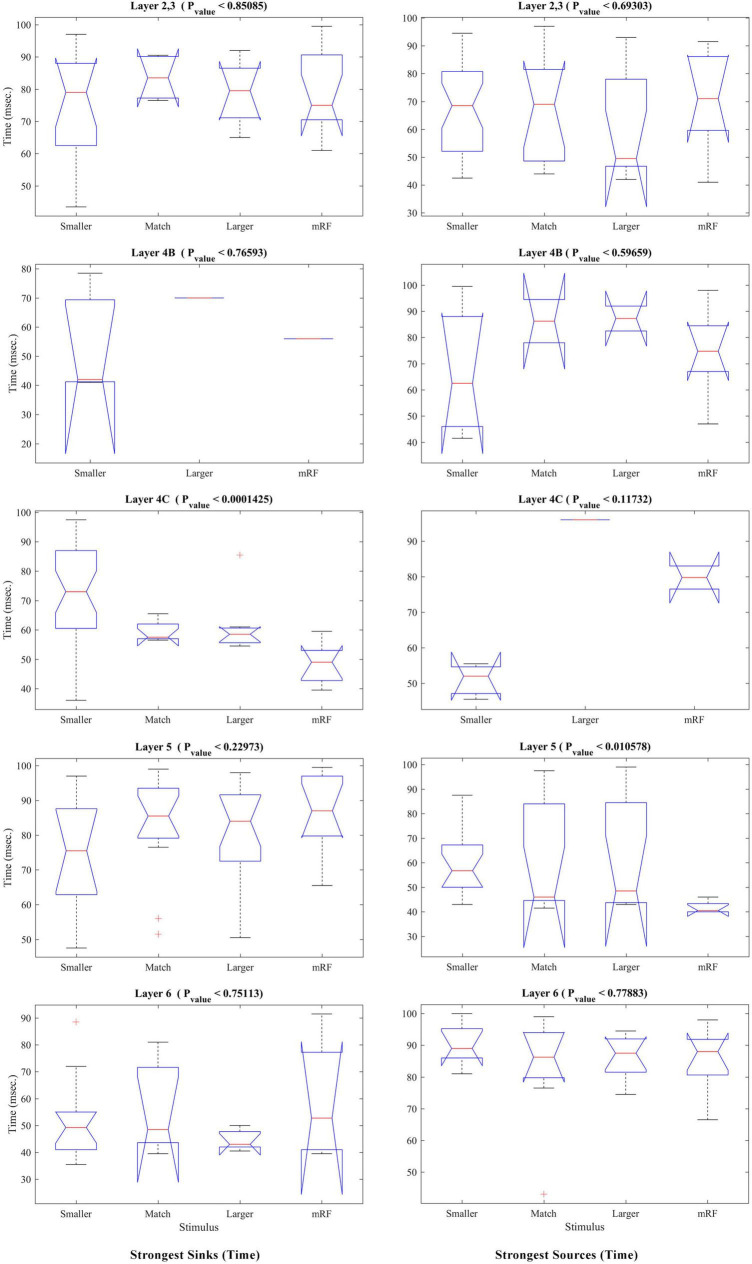
The statistical test for comparing different stimulus conditions in each layer. The Kruskal–Wallis test, which is performed in Figure 6, is analyzed in this figure with the approach of layer separation and comparison of stimulus types. The result of the statistical test of the time of the strongest sinks is shown in the **(left column)** and the time of the strongest sources is shown in the **(right column)**. The type of stimulus is specified on the X-axis. The Y-axis represents the time in milliseconds. At the top of each figure, the layer number and *P*-value are written.

In order to compare the types of stimuli together and to check their differentiation in terms of time and strength of sinks and sources, statistical analysis was performed on the same data, but by comparing the type of stimulus and separately for each layer. The results related to the occurrence time of sinks and sources are shown in Figure 8. Each row represents a layer that is written above it. *P* value is acceptable in layers 4C at the time of the sink and in layer 5 at the time of the source.

**FIGURE 9:**
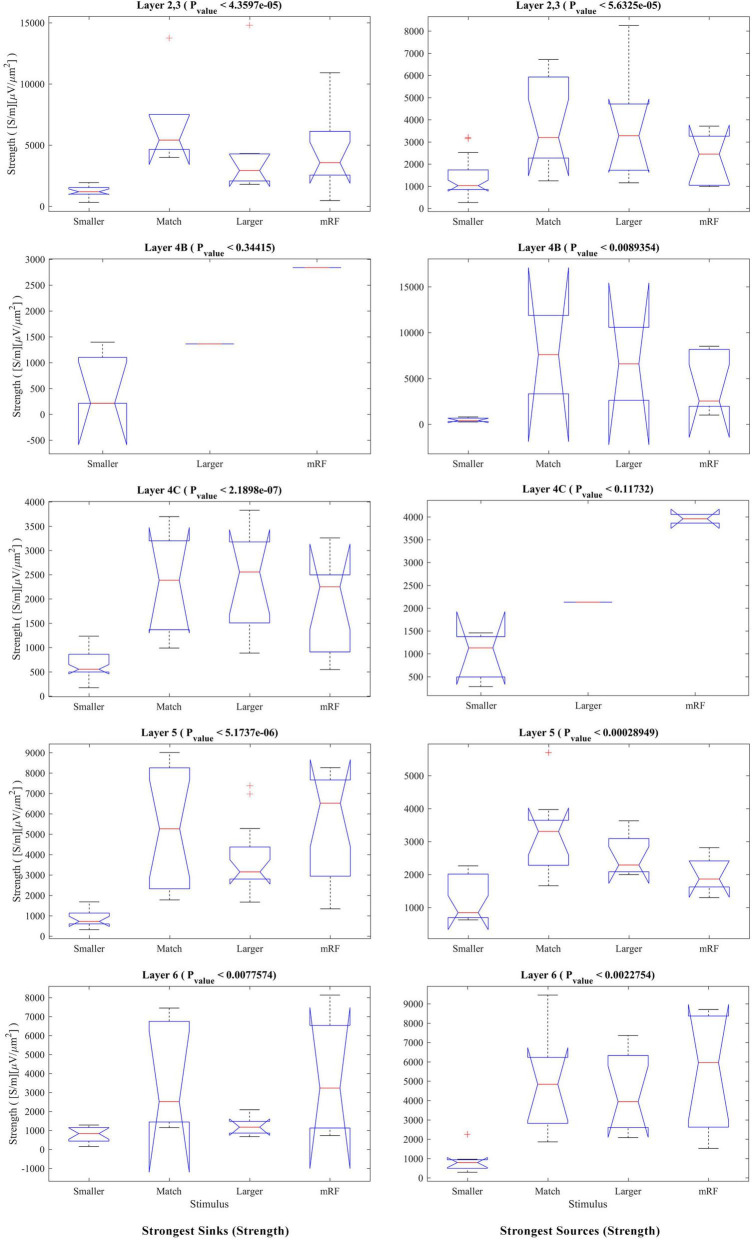
The Kruskal–Wallis test for sink and source strengths based on the type of stimulus. The Kruskal–Wallis test in Figure 7 is shown in this figure with the approach of comparing stimulus types in each layer. The **(left column)** shows the strongest sinks and the **(right column)** shows the strongest sources. The Y-axis expresses the power of the sink or source. Other cases are similar to Figure 8.

In order to investigate the phenomenon of suppression, we need a statistical comparison of the amount of sink and source among the stimulus types separately for each layer. The result of this study in Figure 9 shows that there is a significant difference between all types of stimuli in all layers (except 4B at the sink strength and 4C at the source strength).

In Tables 2–5, the results of the statistical analysis of source time, source strength, sink time and sink strength are presented, respectively. The gray column is the comparison of all layers. And the columns on the right show the comparison of the stimulus equal to the receptive field with the mRF stimulus, the larger stimulus, and the smaller RF stimulus, respectively.

**TABLE 2:** The statistical test in each layer for source time analysis.

Source time test (*P*_*value*_)				
Layers	All stimuli	Match vs.		
		mRF	Larger	Smaller
2,3	0.693	0.7513	0.3633	1
4B	0.5966	0.3173	1	0.5024
4C	0.1173	–	–	–
5	0.0106	0.0231	0.8848	0.2695
6	0.7788	0.9233	0.8587	0.3833

In this table, the results of the Kruskal–Wallis statistical test for the source time analysis are shown separately for each layer. All Stimuli column corresponds to the comparison of all stimuli, which is marked with gray color. In the right columns, the result of the statistical test between the stimulus the match size with receptive field (RF) vs. other stimuli is stated.

**TABLE 3:** The statistical test in each layer for source strength analysis.

Source strength test (*P*_*value*_)				
Layers	All stimuli	Match vs.		
		mRF	Larger	Smaller
2,3	0.00005	0.1351	0.6203	0.0001
4B	0.0089	0.1824	0.4386	0.0455
4C	0.1173	–	–	–
5	0.00029	0.0388	0.2898	0.0003
6	0.0023	0.7728	0.9292	0.0026

In this table, the results of the Kruskal–Wallis statistical test for the source strength analysis are shown separately for each layer. Other descriptions are as in Table 1.

**TABLE 4:** The statistical test in each layer for sink time analysis.

Sink time test (*P*_*value*_)				
Layers	All stimuli	Match vs.		
		mRF	Larger	Smaller
2,3	0.8509	0.3955	0.4649	0.4873
4B	0.7659	–	–	–
4C	0.0001	0.0077	0.5596	0.0202
5	0.2297	0.6083	0.4727	0.1256
6	0.7511	0.9021	0.3472	0.6767

In this table, the results of the Kruskal–Wallis statistical test for the sink time analysis are shown separately for each layer. Other descriptions are as in Table 1.

**TABLE 5:** The statistical test in each layer for sink strength analysis.

Sink strength test (*P*_value_)				
Layers	All stimuli	Match vs.		
		mRF	Larger	Smaller
2,3	0.00004	0.2342	0.0882	0.0012
4B	0.3442	–	–	–
4C	0.00000022	0.3545	0.9578	0.000078
5	0.0000052	0.8073	0.1262	0.000018
6	0.0078	0.8065	0.0758	0.0041

In this table, the results of the Kruskal–Wallis statistical test for the sink strength analysis are shown separately for each layer. Other descriptions are as in Table 1.

**FIGURE 10:**
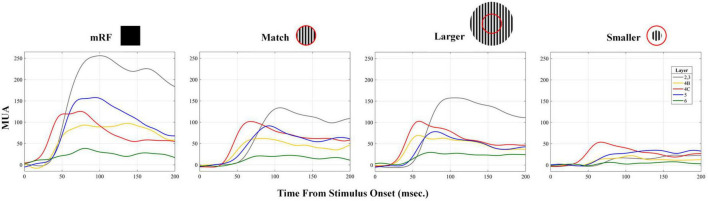
Laminar average multiunit activity (MUA) in response to stimuli. An example of the MUA response averaged in response to all four types of stimuli is shown. The X-axis shows the time in milliseconds and the Y-axis represents MUA. The stimulus type is written at the top of each column. The number of layers is indicated at the top of the first column.

To compare between CSD and multi unit activity (MUA) and check the correlation between them, the average of MUA in each layer was calculated separately for stimulus types. A sample of MUA test is shown in the Figure 10. Each column represents a type of stimulus and each layer is represented by a distinct color. We checked the correlation between the time and the maximum MUA value with the values extracted from CSD (sink and source times and sink and source strengths). Table 6 shows the obtained results separately for each type of stimulus.

**TABLE 6:** The correlation between multiunit activity (MUA) and current source density (CSD) across layers.

Feature Stimulus	MUA (Time) vs.				MUA (Value) vs.			
	Sink time		Source time		Sink strength		Source strength	
	*R*_value_	*P*_value_	*R*_value_	*P*_value_	*R*_value_	*P*_value_	*R*_value_	*P*_value_
mRF	56.9	0.317	−17.7	0.776	17.7	0.775	−81.5	0.092
Match	57	0.425	−48	0.523	−2.8	0.972	−13.6	0.863
Larger	−21.1	0.733	−46.7	0.428	66.6	0.219	−28.1	0.647
Smaller	−13.6	0.828	21.7	0.727	−2.1	0.974	22.1	0.721

The correlations between MUA and CSD across layers are expressed in terms of time and value for stimulus types. The *P*_value_ and *R*_value_ corresponding to each one are written in the table.

**FIGURE 11:**
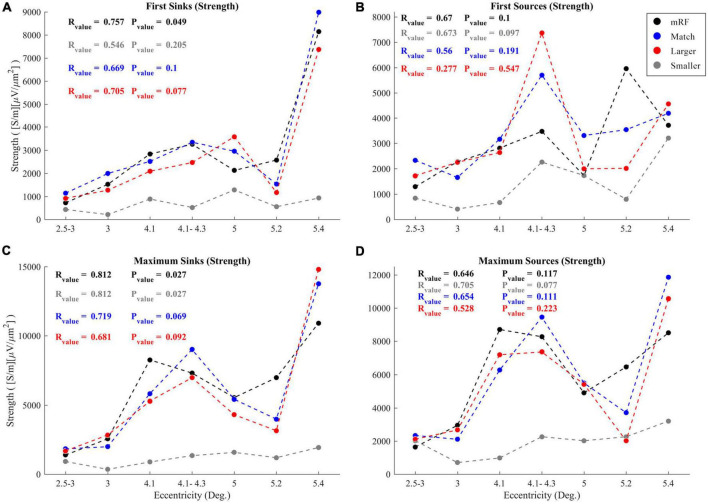
Value of first sinks **(A)** and sources **(B)** in current sinks and sources (CSD) profile in response to black square stimulus (black), equal stimulus size with RF (blue), larger (red), and smaller (gray) than RF with increasing eccentricities. The **(C)** and **(D)** graphs show values of maximum sinks and sources. The R and *P*-values corresponding to the correlation coefficients of all four types of stimuli are written with the legend colors. The horizontal and vertical axes indicate eccentricity and strength of sinks and sources, respectively.

Another investigation was conducted on sinks and sources. To check the trend of changes in sinks and sources with increasing eccentricity, two of the specific cases were examined. These two items include the strengths from the first sink (source) and the maximum sink (source). It was determined that the strength of the first and maximum sinks and sources increased with increasing eccentricity. In Figure 11, the first and maximum sink and source strengths have been plotted against increasing eccentricity. The *P*_value_ and R_value_ corresponding to each stimulus are shown in the graph, each indicated in a color matching with the color of the corresponding graph. This is done to better check the trends.

As for the first and maximum sink and source strengths against increasing eccentricity in all types of stimuli under study, upward trends were observed, though with different *P*_value_ and R_value_. In Figure 11A, the strength of the first sinks with increasing eccentricity, the most ascending trend with the lowest *P*_value_ was related to the black square stimulus (R_value_ = 75.7%, *P*_value_ = 0.049), followed by the stimulus larger than the RF (R_value_ = 70.5%, *P*_value_ = 0.077). The lowest level of ascent was related to the stimulus with a size smaller than RF (R_value_ = 54.6%, *P*_value_ = 0.205). But in Figure 11B related to the first source strengths, the most upward trend with the lowest *P*_value_ was related to the stimulus smaller than RF (R_value_ = 67.3%, *P*_value_ = 0.097). The second place was related to the black square stimulus (R_value_ = 67%, *P*_value_ = 0.1). The lowest upward trend was associated to the stimulus larger than RF (R_value_ = 27.7%, *P*_value_ = 0.547).

Regarding the maximum sink strength in Figure 11C, the most upward trend with increasing eccentricity belonged equally to the black square stimulus and stimulus smaller than RF simultaneously (R_value_ = 81.2%, *P*_value_ = 0.027). The lowest upward trend was related to the stimulus larger than RF (R_value_ = 68.1%, *P*_value_ = 0.092). In the case of the maximum source strength shown in Figure 11D, as with the first sources, the largest upward trend was seen in the stimulus smaller than RF (R_value_ = 70.5%, *P*_value_ = 0.077), while the lowest upward trend was related to the stimulus larger than RF (R_value_ = 52.8%, *P*_value_ = 0.223).

## 4. Discussion

The LFP appears widely different throughout the brain areas. The magnitude of the superficial compartment compared to deep layers was twice as large in spontaneous ongoing activity fluctuations in the gamma frequency range (Maier et al., 2010). Adjusting neurons and intracolumnar coupling in V1 is one of the functions of the thalamus gland. For instance, the thalamocortical loop may control the neurons for the lateral geniculate nucleus (LGN) in layers 4C and 6 (primary target layers) in V1 (Dougherty et al., 2017). If there are visual stimuli, the gamma frequency fast oscillations (30–90 Hz) response is observed. In the absence of this stimulus, slow fluctuations are seen in the primary visual cortex (Berens et al., 2008).

Sinks and sources represent the feedback and feedforward structure of each layer. The origin and target of cortical feedback are neurons in the infragranular layers (Dougherty et al., 2017). The superficial compartment is where efferent projections are mainly directed to extrastriate visual areas. The deep part is efferent projections that are largely directed to the lateral geniculate nucleus, pulvinar, and superior colliculus. The simplified interpretation of LFP activity in the superficial and deep compartments could be considered as its activity in the superficial compartment, related primarily to corticocortical processing, and in the deep one (efferent projections are largely directed to the lateral geniculate nucleus, pulvinar, and superior colliculus) is possibly related to interactions with subcortical structures. Most likely, however, the above explanation is not very accurate because apical dendrites contribute to the supragranular LFP (Maier et al., 2010). A sink and source pattern could be observed at the boundary of granular/infragranular layers (Spaak et al., 2012). Supragranular and granular layers possess the highest density of synapses in macaque V1 (O’Kusky and Colonnier, 1982). Synaptic inputs from different brain regions commonly project to different laminar layers of the local cortex (Martín-Vázquez et al., 2018).

Using CSD analysis, we investigated the effects of changing the type of stimulus on the rate of information arrival and departure. The CSD analysis in studies showed that initial sinks originate from thalamic or intracortical regions. Next sinks were hypothesized to be elicited by transsynaptic intracortical processing. Their other possible generators are repetitive post-discharges originating from the thalamus or inputs from outside of the activation components such as the frontal cortex, the contralateral hemisphere, or the hippocampus (Mitzdorf, 1985). The latter is to determine the higher-order processes, such as learning, cognitive control, and memory, which are the foundation for modeling exact neuronal circuits (Schaefer et al., 2015). The synaptic potential could cause a current sink on the dendritic tree and a current source at the soma (Berens et al., 2008). The primary geniculate input in V1 is detected by a distinct current sink at granular layer 4C. It is thought that this sink is due to the composition of excitatory postsynaptic potentials. The CSD analysis can find the position of this primary retinogeniculate fire of activation (Mitzdorf, 1985).

Current source density patterns are series of sinks and sources. The question arises as: are they all the same? No, because these sinks and sources that can be seen in later times may interfere with each other and are far from reality. Hence, we have to take a critical look at late sinks and sources. Because of accidentally radiating dendritic arbors, the sinks and sources are neutralized. So stellate neurons in layer IV have been believed to contribute little to cortical LFPs (Tanaka and Nakamura, 2019). A local imbalance between the sinks and sources can possibly generate electric potentials. Therefore, we do not know whether the LFPs generated in this way are large enough to appear. We know overlapping current sinks and sources in the non-laminar structures (e.g., the striatum) cannot generate observable LFPs. The current sink on the apical dendrites as well as the current source on the basal dendrite can generate large LFPs. Senzai et al. (2019) observed that in superficial layers, the primary sink was accompanied by a source and a sink-source pair corresponding to the border between the sink in layers 5 and 6 and the source in layer 6. Visual stimulation also created a primary sink between layers 3 and 4, which probably reflected afferent activation from the lateral geniculate body. Collaterals of thalamocortical afferent axons innervate both superficial and deep layers in contact with several types of neurons. However, the highest compression of thalamocortical afferents exists in layer 4.

The time and CSD strength for the strongest sinks and sources for all types of stimuli considered are extracted. The time of the sinks in the four types of stimuli states, namely, the 0.5 black square, smaller than RF, equal to, and larger than RF was observed. By observing the graph related to the time of occurrence of sinks, it was found that the initial sinks occurred in all stimuli in layers 6 and 4C. Then, sinks were situated in layers 2/3, and 5. The primary input layers are 4C and 6, and the secondary input layers are 2/3, and 5. In layer 4C, the black square stimulus received the first sinks, followed by stimuli equal to and larger than the receptive field. In the case of the stimulus smaller than the receptive field, as observed, a wide time range includes the time of occurrence of its sinks. Unlike layer 4C, in layer 6, almost all types of stimuli presented in this study had sinks at almost the same time.

Layer 4C (input layer) only has local recurrent connections and feedforward projections from the lateral geniculate nucleus (LGN). Layers 2/3, and 4B (output layers) have rich horizontal and feedback connections (Wang et al., 2020; Yang et al., 2022). The earliest current sink of CSDs referred to the middle of layer 4Ca (Mitzdorf and Singer, 1979). Feedforward inputs terminate in layer 4 (dominating in supragranular layers) of sensory cortical areas, then go to layers 2/3, and then onward to layers 5 and 6, and from there they pass on to layers 2/3. In contrast, feedback connections from downstream areas terminate in layers 1 and 5, and then pass on to layers 2/3 (dominating in supragranular layers). The stimuli surrounding the receptive field, believed to affect the processing in V1 primarily through feedback projections, first activate V1 in the upper superficial layers and then in layer 5 (Bijanzadeh et al., 2018).

Our analysis related to the time of occurrence of sources showed that the first sources occurred in layers 5, 2, and 3, followed by layers 4B and 6. In fact, the input of primary information is observed first in layers 4C and 6, while the output of primary information is observed in layers 5, 2, and 3. The input of secondary information is first observed in layers 5, 2, and 3, while the output of secondary information is observed in layers 6 and 4B. We showed the information projection with four different types of stimuli quite well.

An important role in the integration of ipsilateral and contralateral inputs can be indicated by the location of the sink in the main thalamic input (layers III/IV) (Schaefer et al., 2015). Studies demonstrated that the feedforward input comes from the lateral geniculate nucleus (LGN) and arrives in layer 4C with a weaker input into layer 6, but feedback connections come from higher visual areas and arrive in layers 1, 2, and 5, avoiding layer 4 (van Kerkoerle et al., 2014). In V1, layer 5 neurons according to the anatomical alignment use a strong control over neurons within the same cortical column. Projections from layer 5 to layers 2/3 organize one of the widest interlaminar projections within the cortical microcircuit (Dougherty et al., 2017). Projections from layer 5 neurons to superficial layers *via* interneurons use an inhibitory effect (Dougherty et al., 2017). The CSD analysis determines synaptic activations at somatic or dendritic levels spatially and temporally (Schaefer et al., 2015). The primary current sink coincides with the first activation from thalamocortical afferents (Gieselmann and Thiele, 2022).

In the present research, investigations were done on the effects of changing the type of stimulus and changing the eccentricity on the flow of information input and output. The boundaries that show the sinks actually show the incoming data. This indicates how much the signal is spread in that place. This issue can be related to the visual expansion of the signal. Distant neuronal activity and horizontal connections propagation within the cortex drive local synaptic activity (Gawne, 2010). Different studies of LFP propagation have different results. Some of them report a few hundred microns (Engel et al., 1990; Katzner et al., 2009; Xing et al., 2009). Others researchers confirmed up to several millimeters (Mitzdorf, 1985; Logothetis et al., 2001; Kreiman et al., 2006). Kajikawa and Schroeder (2011) showed the spread of the LFP laterally well outside the 200∼400 μm range, with a vertical spread increasing by several millimeters beyond the auditory cortex. Berens et al. (2008) guessed LFP gamma-band reflects signals from an area measuring at least 500–800 μm in diameter.

## 5. Conclusion

In this study, we used LFP data from the primary visual cortex of non-human primate electrophysiology recording to show that neuronal responses are distinguishable responding to different stimulus properties like size and type. We used CSD function in different V1 layers and different eccentricities to justify the main hypothesis of the paper, which is the quantification of input and output currents to V1.

We obtained a map of the LFP (created by using CSD) with changes in stimulus type as well as increased eccentricity in the Primary Visual Cortex. By extracting the time and strength of sinks and sources we detected the response changes of neural population activity across cortical layers. When the stimulus was smaller than the RF’s size, the CSD mapping was not clear. But in three other stimulus types (black square, larger and optimized stimulus size), the response changes were distinguishable. Carefully in the LFP results, we saw that the compression of the graph (the distance between the sink and the source) decreases with the increase of the eccentricity. Of course, accurate analysis is suggested for this case. We used CSD instead of raw LFP response because of the noise and variations in LFP response.

The time of CSDs showed that first, the sinks occurred in layers 4C and 6 (input of information) and the sources in layers 2/3, and 5 (output of information), then conversely the sinks occurred in layers 2/3, and 5 and the sources in layers 4 and 6.

In terms of the strength of sinks and sources, the weakest sinks and sources in all layers are observed in response to stimulus smaller than RF. In general, the response of the stimulus larger than RF is weaker than the other two stimuli (MRF and equal to RF) which indicated the suppression phenomenon. The statistical test was performed both in terms of comparing the types of stimulus in each layer and in terms of comparing the layers in each stimulus.

Additionally, with the increased eccentricity (2–6 degrees), sink’s and source’s strengths were increased as well as across cortical layers.

Our study focused on changes in LFP polarity across layers and eccentricity with changes in stimulus size. LFP polarity changes in the brain can be helpful in BCI chip implants as they provide a measure of neural activity. The LFP signals can reflect changes in the synaptic activity, ionic currents and action potentials, and the changes in polarity can indicate changes in the direction of the current flow within the tissue. By monitoring the LFP polarity changes, the BCI chips can better understand the underlying neural activity and make more accurate predictions about the user’s intent, which can improve the performance of BCI systems. This can lead to more effective communication between the brain and the BCI devices, enabling people with disabilities to control devices and communicate more effectively.

## Data availability statement

The original contributions presented in this study are included in the article/supplementary material, further inquiries can be directed to the corresponding authors.

## Ethics statement

All experimental procedures were in accordance with protocols approved by the University of Utah Institutional Animal Care and Use Committee and with NIH guidelines.

## Author contributions

FK conceived and designed the analysis, contributed to analysis tools, performed the analysis, and wrote the manuscript. SS and RL conceived and designed the analysis, contributed to analysis tools, performed the analysis, wrote the manuscript, and supervised the study. MD contributed to analysis tools and performed the analysis. All authors contributed to the article and approved the submitted version.
